# Comparative molecular detection and phylogenetic analysis of *Babesia canis vogeli* in naturally infected dogs using two *18S rRNA* primer sets in Khon Kaen, Thailand

**DOI:** 10.14202/vetworld.2025.2663-2677

**Published:** 2025-09-11

**Authors:** Clara Ancilia Pramita Kusumasri, Patchara Phuektes, Numfa Fungbun

**Affiliations:** 1 Faculty of Veterinary Medicine, Khon Kaen University, Khon Kaen, Thailand; 2 Division of Pathobiology, Faculty of Veterinary Medicine, Khon Kaen University, Khon Kaen, Thailand; 3 Division of Companion Animal Medicine, Faculty of Veterinary Medicine, Khon Kaen University, Khon Kaen, Thailand

**Keywords:** *18S rRNA*, *Babesia canis vogeli*, canine babesiosis, phylogenetic analysis, polymerase chain reaction, Thailand

## Abstract

**Background and Aim:**

Canine babesiosis, primarily caused by *Babesia canis vogeli* in Thailand, is a significant tick-borne disease of veterinary concern. Molecular diagnostics targeting the *18S rRNA* gene have enhanced detection sensitivity and specificity compared to conventional methods. This study aimed to identify and characterize *B. canis vogeli* in naturally infected dogs in Khon Kaen, Thailand, to compare the diagnostic performance of two primer sets (Bab7/Bab9 and Babf/Babc), and to perform phylogenetic analysis of the isolates.

**Materials and Methods:**

A total of 159 ethylenediaminetetraacetic acid blood samples from client-owned dogs presented to the Veterinary Teaching Hospital, Khon Kaen University, between July and October 2024, were examined. Samples underwent Giemsa-stained blood smear microscopy and PCR amplification of the *18S rRNA* gene using both primer sets. Positive amplicons were sequenced and analyzed phylogenetically using the Maximum Likelihood method. Limit of detection (LOD), sensitivity, specificity, and positive predictive value (PPV) were calculated for each primer set using sequence-confirmed results as the reference.

**Results:**

Microscopy detected *B. canis* in 19/159 (11.9%) of samples, while PCR increased detection to 23/159 (14.47%). Babf/Babc detected all positive cases (100% sensitivity), while Bab7/Bab9 detected 95.65% of positives. Both primer sets achieved 100% specificity and PPV, with an equal LOD of 10^5^ DNA copies. Bab7/Bab9 also amplified *Hepatozoon canis* at a distinct amplicon size (503 base pair). Sequence analysis confirmed all *Babesia*-positive samples as *B. canis vogeli*, showing 96.34%–100% identity with global isolates. Phylogenetic analysis grouped the sequences with *B. canis vogeli* from multiple geographic regions, revealing minimal intraspecific variation.

**Conclusion:**

*B. canis vogeli* was the only subspecies identified in naturally infected dogs in Khon Kaen during the study period. Babf/Babc demonstrated superior diagnostic sensitivity for *B. canis vogeli*, whereas Bab7/Bab9 offered broader detection, including *H. canis*. Phylogenetic analysis revealed close genetic relationships with isolates worldwide. These findings support the use of Babf/Babc for specific diagnosis and Bab7/Bab9 for broader screening in endemic regions.

## INTRODUCTION

Canine babesiosis is a globally significant tick-borne disease of dogs caused by intraerythrocytic protozoa of the genus *Babesia* [1–3]. These parasites are morphologically identified in blood smears by their trophozoite and merozoite stages, appearing either as large piroplasms (*Babesia canis*; 3–5 μm) or small piroplasms (*Babesia* gibsoni; 1.5–2.5 μm), typically pear- or ring-shaped within erythrocytes. *B. canis*, the most common large *Babesia* species, is classified into three subspecies, *B. canis vogeli*, *B. canis canis*, and *B. canis rossi*, based on phylogenetic, vector, pathogenicity, and cross-immunity differences. These are now recognized as distinct species [4–8]. Of these, *B. canis vogeli* is generally the least pathogenic, while *B. canis rossi* is the most virulent and is associated with severe disease outcomes [7, 9]. Clinical presentation ranges from asymptomatic to severe illness, depending on parasite virulence and host immunity. Common signs include fever, anorexia, lethargy, anemia, thrombocytopenia, splenomegaly, and jaundice [10, 11]. Prevalence in Thailand varies widely, 2.5% in central regions, 19.9% in the south, and province-specific rates of 19.5% in Khon Kaen, 6.3% in Mahasarakham, and 2.8% in Songkla [12–16]. *B. canis vogeli* predominates in Thailand, likely due to the widespread distribution of *Rhipicephalus sanguineus*, the principal tick vector infesting dogs across Asia [7, 17, 18]. Diagnostic approaches include microscopy of stained blood smears, which remains the conventional method but suffers reduced sensitivity in low-parasitemia or early infection. Serological assays such as immunofluorescent antibody testing provide additional options but are limited by low antibody levels during acute infection. In contrast, molecular techniques, particularly polymerase chain reaction (PCR), offer superior sensitivity and specificity, enabling detection even in subclinical cases and facilitating accurate species identification through DNA sequencing [19–22]. Genetic markers form the basis of molecular detection, with reported targets including cytoplasmic protein 29 kDa, thrombospondin-related adhesive protein [23], 18S rRNA, 5.8S rRNA, 28S rRNA [24], heat shock protein 70, β-tubulin, internal transcribed spacers (ITS), and cytochrome c oxidase 1 [20, 25]. The *18S rRNA* gene is the most widely used marker for piroplasm detection and diversity studies by Paulino *et al*. [23] and Hrazdilová *et al*. [26] due to its highly conserved regions interspersed with variable domains. This structure makes it suitable for species identification, phylogenetic analysis, and resolving relationships at higher taxonomic levels [24, 26–29]. Previous work has shown that the Babf/Babc primer set (Kordick’s *Babesia* common) achieves 100% sensitivity and specificity for *B. canis vogeli* detection in a cohort of 55 dogs [30]. The Bab7/Bab9 primer set is broader in scope, targeting all *Babesia* species, as confirmed by Basic Local Alignment Search Tool (BLAST) analysis (the National Center for Biotechnology Information) [31]. However, a direct comparison of these primer sets for detecting *B. canis vogeli* in naturally infected dogs from Khon Kaen, Thailand, has not yet been conducted.

Although canine babesiosis has been reported across Thailand, with *B. canis vogeli* as the predominant etiological agent, data from the northeastern region, particularly Khon Kaen, are limited to prevalence surveys using microscopy or single molecular protocols. While molecular detection of the *18S rRNA* gene has proven reliable for *Babesia* identification, few studies have systematically compared different primer sets for diagnostic accuracy in naturally infected dogs under field conditions in this region. The Babf/Babc primer set has been validated for high specificity toward *B. canis vogeli*, whereas the Bab7/Bab9 primer set targets a broader range of *Babesia* species. However, their relative diagnostic performance, detection limits, and potential for cross-amplification of co-circulating haemoparasites such as *Hepatozoon canis* have not been evaluated side-by-side in an endemic, mixed-infection environment. Furthermore, phylogenetic data for *B. canis vogeli* from Khon Kaen are scarce, limiting understanding of the genetic relatedness of local isolates to global strains. This knowledge gap constrains both accurate diagnosis and broader epidemiological insights needed for vector-borne disease control in companion animals.

This study aimed to (1) detect and molecularly identify *B. canis vogeli* in naturally infected dogs from Khon Kaen, Thailand, using *18S rRN*A gene amplification with two primer sets (Bab7/Bab9 and Babf/Babc); (2) compare the diagnostic performance of these primer sets in terms of sensitivity, specificity, positive predictive value (PPV), and limit of detection (LOD); (3) assess their potential cross-reactivity with other haemoparasites such as *H. canis*; and (4) perform phylogenetic analysis of *B. canis vogeli* isolates to determine their genetic relationships with reference sequences from different geographic regions. The findings are expected to inform the selection of optimal molecular tools for accurate diagnosis, contribute to regional surveillance data, and enhance understanding of the molecular epidemiology of *B. canis vogeli* in Thailand.

## MATERIALS AND METHODS

### Ethical approval

This study was approved by the Institutional Animal Care and Use Committee of Khon Kaen University, in accordance with the Ethics of Animal Experimentation guidelines of the National Research Council of Thailand (Reference No. 660201.2.11/414(67); Date May 23, 2024), and USDA animal care. All dog owners provided informed consent.

### Study period and location

This study was conducted from July 2024 to January 2025 at the Veterinary Diagnostic Laboratory, Veterinary Teaching Hospital, Faculty of Veterinary Medicine, Khon Kaen University, Khon Kaen, Thailand.

### Animals

**Figure 1:**
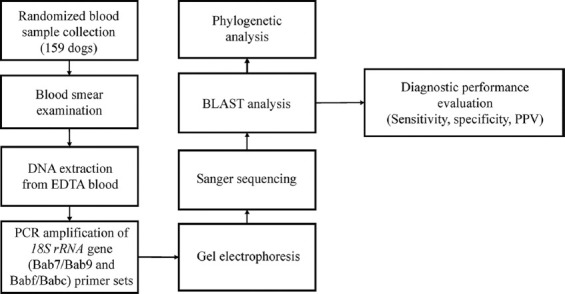
Schematic overview of the study methodology.

A total of 159 ethylenediaminetetraacetic acid (EDTA) blood samples were randomly collected from client-owned dogs presented to the Veterinary Teaching Hospital, Faculty of Veterinary Medicine, Khon Kaen University, Thailand, for routine checkup or general clinical diagnosis. The exclusion criteria were emergency and critical cases (such as hypovolemic shock or acute respiratory distress syndrome or end-stage chronic kidney, heart, or liver diseases). Signalment (breed, age, and sex), clinical signs, and other relevant clinical data were recorded for each dog. The dogs had several ages, from young puppies at 3 months old to senior dogs at 15 years old. Of these, 97 were male and 62 were female. Regarding breed, 93 dogs (58.5%) were purebred and 66 (41.5%) were mixed breed. Figure 1 shows an overview of the study workflow.

### Blood sample collection and blood smear examination

Approximately 1–2 mL of blood was collected from either the cephalic or saphenous vein of each dog into sterile EDTA vacutainer tubes for both blood smear examination and molecular analysis. The EDTA blood samples were maintained at room temperature (25^o^C–30^o^C) before the routine blood smear. A blood smear was performed using 10% Giemsa stain for 3 min. The blood smears were examined at 1,000× magnification under a light microscope (Olympus CX31, Olympus Corporation, Japan) by a well-trained and experienced laboratory staff to identify the morphological features of blood parasites. The EDTA-blood samples were then stored at −80°C until DNA extraction and PCR analysis.

### DNA extraction

Genomic DNA was extracted from 200 μL of each EDTA-blood sample using a commercial DNA extraction kit (Thermo Scientific, Lithuania; Lot No. 01027389), following the manufacturer’s instructions. DNA was eluted in a final volume of 100 μL of EB. DNA quality was assessed using an A260/A280 ratio of ~1.8. The DNA concentration was measured using a BioDrop Duo spectrophotometer (BioDrop Ltd., UK). The extracted DNA samples were stored at −80°C until use in PCR assays.

### PCR amplification of *18S rRNA* gene

PCR amplification was performed in a total reaction volume of 20 μL. The reaction mixture contained 10 μL of DreamTaq Green PCR Master Mix (2X; Thermo Scientific, Waltham, MA, USA), which included DreamTaq DNA polymerase, reaction buffer, 0.4 mM of each dNTP, and 4 mM MgCl_2_. In addition, 0.4 μL of each primer (5 pmol), and 2 μL of genomic DNA template were added. DNase-free water was added to bring the final reaction volume to 20 μL. PCR reactions were performed in duplicate. The amplicon sizes were verified using a 100-base pair (bp) marker.

The primer targeting the *18S rRNA* gene was validated by Duarte *et al*. [30] and Martin *et al*. [31]. The two primer sets of the *18S rRNA* gene and the nucleotide sequences of the two primer sets are listed in Tables 1 [30, 31] and 2.

**Table 1:** Two primer sets of the *18S rRNA* gene.

Primer	Amplified region (bp)	Annealing temperature (°C)	Reference
Babf (F) Babc (R)	394	55	[30]
Bab7 (F) Bab9 (R)	490	56	[31]

F = Forward, R = Reverse, bp = Base pair

**Table 2:** The nucleotide sequences of the two primer sets and the GenBank reference.

Primer	Primer sequence (5’ - 3’)	GenBank reference	Position
Bab7 (F) Bab9 (R)	GGCTACCACATCTAAGGAAG CTAAGAATTTCACCTCTGACAG	*B. canis vogeli* (AY072925)	369–388 809–831
Babf (F) Babc (R)	AAGTACAAGCTTTTTACGGTG CCTGTATTGTTATTTCTTGTCACTACCTC	*B. canis vogeli* (AY072925)	61–81 425–453

F = Forward, R = Reverse

### PCR conditions

The PCR conditions were adapted from the protocols described by Duarte *et al*. [30] and Martin *et al*. [31]. The optimal annealing temperature was validated by performing the annealing at various gradient temperatures for the Bab7/Bab9 and Babf/Babc primer sets. The thermal cycling program consisted of an initial denaturation at 95°C for 3 min, followed by 30 cycles of denaturation at 95°C for 1 min, annealing at 56°C (Bab7/Bab9) or 55°C (Babf/Babc) for 30 s, and extension at 72°C for 1 min, with a final extension at 72°C for 5 min. Amplification was performed using a T100 Thermal Cycler (Bio-Rad, Hercules, CA, USA).

Plasmid DNA cloned from *Babesia* amplified by each primer set was used as a positive control and was included in all PCR tests. A negative control (no template DNA) was included in each PCR run.

### Amplicon detection and visualization

All PCR products were visualized by electrophoresis in a 1.5% agarose gel containing RedSafe dye (iNtRON Biotechnology, Korea) and observed under ultraviolet light using a Gel Doc XR+ system (Bio-Rad). Electrophoresis was performed at 100 V for 30 min in 1X TBE buffer. A 100 bp DNA ladder (VC 100 bp Plus DNA Ladder, Vivantis Technologies, Malaysia) was used as a molecular weight marker. The gels were observed under ultraviolet light using a Gel Doc XR+ imaging system (Bio-Rad) and Image Lab software (Bio-Rad).

### Cloning of the plasmid DNA

Positive PCR products of *18S rRNA* gene amplification, confirmed by sequencing as identical to *Babesia* sequences in the National Center for Biotechnology Information (NCBI, USA) database, were used for plasmid cloning. The GF-1 AmbiClean Kit (Vivantis Technologies) was used to purify PCR products. Ligation was performed using a 1:3 vector-to-insert molar ratio with the pTG-19T PCR Cloning Vector Kit (Vivantis Technologies) in a thermal cycler at 22°C for 3 h.

The ligation mixture was transformed into *Escherichia coli* strain TOP10 competent cells (Invitrogen) through heat shock and cultured overnight at 37°C on Luria-Bertani (LB) agar plates containing 100 μg/mL of ampicillin. Blue-white colony screening was performed to identify recombinant colonies, which were then cultured for plasmid extraction. The colony was selected based on ampicillin resistance. Individual colonies were picked and inoculated into 5 mL of LB broth containing 100 μg/mL ampicillin and cultured overnight at 37°C in a shaking incubator. The plasmid DNA was extracted using a GF-1 Plasmid DNA Extraction Kit (Vivantis Technologies) and quantified using a BioDrop Duo spectrophotometer (BioDrop Ltd.).

The plasmid DNA from each primer set was sequenced and subjected to bidirectional Sanger sequencing (U2Bio Co. Ltd., Seoul, South Korea) using an Applied Biosystems model 3730XL DNA Analyzer. The cloned plasmid DNA amplified using the Bab7/Bab9 primer set showed 99.77% identity with *B. canis vogeli* (GenBank accession no. MN823219.1), while that from the Babf/Babc primer set showed 99.27% similarity with *B. canis vogeli* (GenBank accession no. MT386936.1).

### Sequencing of the PCR products

The PCR products that yielded positive results were purified using the GF-1 AmbiClean Kit (Vivantis Technologies) according to the manufacturer’s protocol. Purified DNA was eluted in DNase-free water, spectrophotometrically quantified, and stored at −20°C until sequencing.

The purified DNA was subjected to unidirectional Sanger sequencing using the forward primer of each primer set and the Sanger dideoxy method (U2Bio Co. Ltd., Seoul, South Korea) on an Applied Biosystems model 3730XL DNA Analyzer. The resulting *18S rRNA* sequences were compared with reference sequences in GenBank using the BLAST tool on the National Center for Biotechnology Information website to confirm species identity.

### Phylogenetic analysis

To examine the relationships between positive samples and *Babesia* *18S rRNA* reference sequences from GenBank, multiple sequence alignment and phylogenetic analysis were performed using MEGA X software v10.2.6 (https://www.megasoftware.net/). The sequences were aligned using the Clustal W algorithm (DNASTAR, Inc., USA). Phylogenetic trees were constructed using the Maximum Likelihood (ML) method based on the Kimura two-parameter model. The tree branching reliability was assessed using bootstrap analysis with 1,000 replications [17].

The final phylogenetic tree based on the Bab7/Bab9 primer set included 37 nucleotide sequences and 319 aligned positions. The Babf/Babc-based phylogenetic analysis included 38 nucleotide sequences, with 301 final alignment positions. All sequences from the positive sample detected by each primer set were included in the respective phylogenetic analyses. In addition, 17 reference sequences of *Babesia* spp. from different geographical regions and species representations were retrieved from the NCBI database using their GenBank accession numbers as follows: *B. canis vogeli*: Brazil (MN823219, EU436752), France (AY072925), USA (AY371198), Egypt (AY371197), China (KJ939326), India (PP859306, MG050158), Thailand (KF621061), Japan (DQ111766); *B. canis canis*: China (MK571835), Romania (HQ662634), Croatia (AY072926); *B. canis rossi*: Africa (KY463430), Turkey (MK918605); and *B. gibsoni*: India (MN134517), China (KP666155). The phylogenetic trees were rooted using *P. falciparum* (GenBank accession no. M19172) as an outgroup in the phylogenetic tree.

### LOD assessment

The LOD for each primer set was assessed by detecting the lowest concentration of *Babesia* DNA using a ten-fold serial dilution ranging from 10^10^ to 10^1^ copies. Plasmid concentration was measured using a BioDrop Duo spectrophotometer. The lowest dilution yielding a visible band on gel electrophoresis was considered the detection limit. The plasmid copy number was calculated as follows [32]:

Copy number = (Plasmid concentration [ng/µL] × 6.022 × 10^23^)/(Plasmid length [bp] × 1 × 10^9^ × 660)

The PCR reactions and thermal conditions for each primer set were performed according to the previously described protocol. The LOD was verified by separate runs.

### Diagnostic performance evaluation

The sensitivity, specificity, and PPV for each primer set were calculated using the following formulas:

Sensitivity (%) = (TP/[TP + FN]) × 100

Specificity (%) = (TN/[TN + FP]) × 100

PPV (%) = (TP/[TP + FP]) × 100

Where:

- TP = True positive: A band appears at the expected size and is confirmed as *Babesia* by sequence alignment with the National Center for Biotechnology Information database.
- FN = False negative: The primer set detected no band, although another primer set confirmed *Babesia* positivity by sequencing.
- FP = False positive: A band appears, but the sequence does not match *Babesia* in the National Center for Biotechnology Information database.
- TN = True negative: No band is observed for the sample with either primer set, and no *Babesia* sequence is detected.
- (Note: The expected amplicon sizes were 490 bp for Bab7/Bab9 and 394 bp for Babf/Babc.)

### Statistical analysis

Categorical variables, including sex, breed, and age, are presented as percentages. Sensitivity, specificity, and positive predictive value (PPV) were expressed as percentages. The sensitivity and specificity confidence intervals (CIs) were calculated using the exact Clopper–Pearson CIs. CI for the PPV was calculated using a standard logit model. MedCalc statistical software (MedCalc Software Ltd., USA) was used for CI calculation.

## RESULTS

### Clinical data

*Babesia* infection was detected in 23/159 dogs (14.47%), ranging in age from 3 months to 15 years. Notably, 9/23 (39.13%) of positive cases occurred in puppies aged ≤1 year, and 7/23 (30.43%) in senior dogs aged >7 years. Infections were more common in males (13/23, 56.52%) than in females (10/23, 43.48%).

The study population included mixed-breed and purebred dogs, indicating a broad host susceptibility to the parasite. Mixed-breed dogs were the most commonly affected (10/23, 43.48%). Each of the following purebred breeds, Poodle, French Bulldog, Pomeranian, and American Bully, accounted for two cases each (2/23, 8.69%), while other breeds made up 1/23 (4.35%) of the cases.

Of the 23 *Babesia*-positive dogs, one was diagnosed solely with babesiosis, whereas the remaining 22 had concurrent illnesses or coinfections with *Ehrlichia canis* and *Anaplasma platys*.

The clinical signs among *Babesia*-positive dogs were variable. The dog diagnosed solely with babesiosis showed no clinical signs, and its hematology and biochemistry results were within normal ranges. Some co-infected dogs exhibited clinical abnormalities such as weakness, anorexia, lethargy, pyrexia, anemia, and thrombocytopenia.

### Microscopic examination

Microscopic examination identified *B. canis* in 19/159 samples (11.95%), characterized by large piroplasms within red blood cells (RBCs).

### PCR amplification and nucleotide sequence analysis

PCR assays improved the detection rate to 23/159 samples (14.47%). The Bab7/Bab9 primer set yielded 22 positive samples, whereas the Babf/Babc primer set yielded 23 positive samples. The expected amplicon sizes were 490 bp for Bab7/Bab9 and 394 bp for Babf/Babc.

BLAST analysis of *18S rRNA* gene sequences from all positive samples revealed high similarity with sequences of *B. canis vogeli* in GenBank. Multiple sequence alignment of *B. canis vogeli* isolates detected by Bab7/Bab9 primers showed 92.88%–100% similarity among local samples and 96.74%–100% identity with *B. canis vogeli* sequences from Brazil, Paraguay, and Taiwan (accession numbers: MN823219, MF459002, MH100718, and HQ148664). For the Babf/Babc primer set, sequences showed 96.68%-100% intra-sample identity and 96.34%–99.73% identity with *B. canis vogeli* from Brazil, India, and Taiwan (accession numbers: MF281424, EU436752, MG050158, and HQ148664).

The results of the microscopic examination and PCR assay, along with the percentage identity of partial sequences of *B. canis* subspecies for each primer set in NCBI, are shown in Table 3.

**Table 3:** Results of microscopic examination and PCR assay with the percentage identity of partial sequences of *B. canis* subspecies in each primer set from NCBI.

No.	Microscopic examination	PCR assays (% identity to *B. canis* *vogeli*)*		Closest accession number in NCBI

		Bab7 and Bab9	Babf and Babc	
1	+	+ (100)	+ (98.90)	MN823219, MF281424
2	+	+ (100)	+ (99.72)	MN823219, MG050158
3	+	+ (100)	+ (99.43)	MN823219, MG050158
4	+	+ (100)	+ (98.90)	MN823219, MF281424
5	+	+ (98.64)	+ (96.34)	MH100718, MG050158
6	+	+ (99.76)	+ (99.73)	MH100718, MF281424
7	+	+ (100)	+ (99.44)	MH100718, MG050158
8	+	+ (96.74)	+ (99.15)	MF459002, EU436752
9	+	+ (100)	+ (99.43)	MN823219, EU436752
10	+	+ (99.53)	+ (98.63)	MN823219, EU436752
11	+	+ (99.29)	+ (99.15)	HQ148664, HQ148664
12	+	+ (99.07)	+ (98.90)	MN823219, EU436752
13	+	+ (100)	+ (99.18)	MH100718, EU436752
14	+	+ (99.76)	+ (99.44)	MH100718, EU436752
15	+	+ (99.52)	+ (99.15)	MH100718, EU436752
16	+	+ (99.29)	+ (98.90)	MH100718, EU436752
17	+	+ (99.53)	+ (99.44)	MH100718, EU436752
18	+	+ (99.76)	+ (99.44)	MN823219, MF281424
19	+	+ (99.76)	+ (99.43)	MN823219, MF281424
54	-	+ (100)	+ (98.90)	MN823219, MF281424
80	-	+ (99.77)	+ (98.62)	MN823219, MF281424
119	-	+ (100)	+ (99.44)	MN823219, MF281424
142	-	-	+ (99.72)	MG050158
n = 159	19/159 (11.95%)	22/159 (13.83%)	23/159 (14.47%)	

Data are presented as positive (+) or negative (-) results.

* All positive PCR results using two primer sets of Bab7/Bab9, and Babf/Babc are closely identical to those of the *B. canis vogeli* subspecies. PCR = Polymerase chain reaction, NCBI = National Center for Biotechnology Information

**Figure 2:**
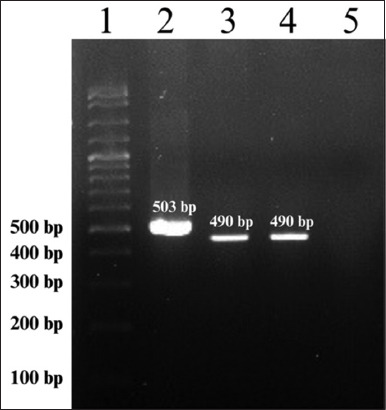
Molecular detection of *Babesia canis vogeli* and *Hepatozoon canis* by *18s rRNA* gene using the Bab7/Bab9 primer set and gel electrophoresis; Lane 1 = DNA ladder (100 base pair [bp]), Lane 2 = Positive *H. canis* at 503 bp (sample number 52), Lane 3 = Positive *B. canis vogeli* at 490 bp (sample number 54), Lane 4 = Positive control, Lane 5 = Negative control.

In addition, the Bab7/Bab9 primer set can amplify *Hepatozoon canis* (503 bp), which closely resembles the 490 bp target band of *B. canis*. In this study, *H. canis* infection was identified in 20/159 (12.58%) dogs using the Bab7/Bab9 primer set and confirmed by sequence analysis. These findings confirm that the two species can be differentiated based on their respective amplicon sizes (Figure 2).

### Phylogenetic analysis

Phylogenetic analysis was conducted on all *B. canis vogeli*-positive sequences from each primer set using the ML method, incorporating reference sequences from GenBank representing different regions and species. Positions containing alignment gaps or missing data were excluded using a 95% partial deletion threshold.

**Figure 3:**
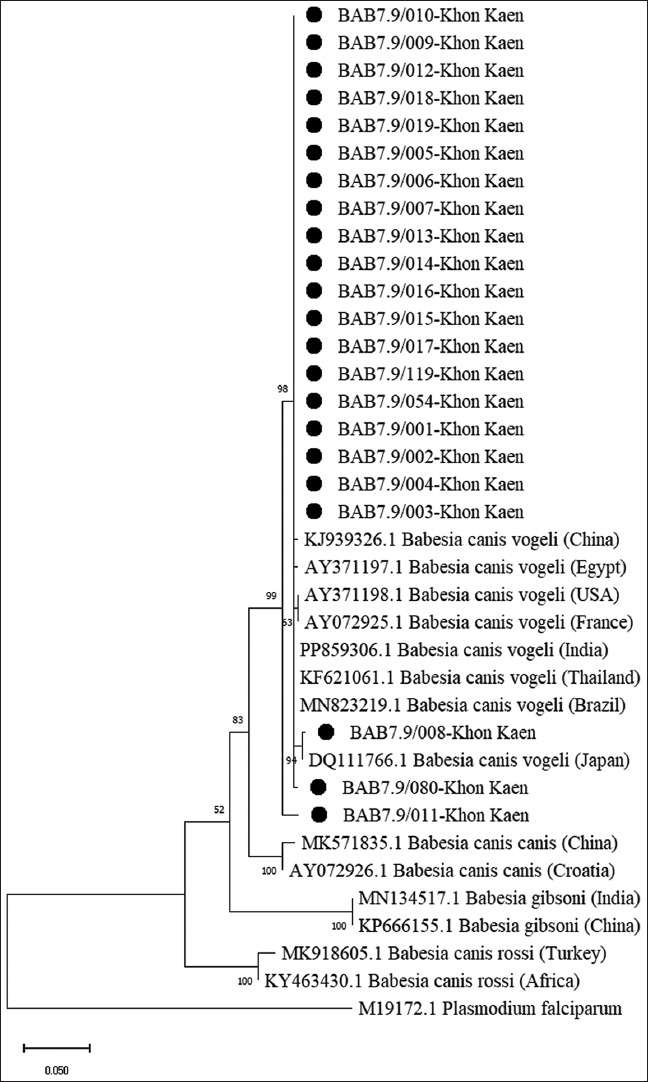
Phylogenetic tree of *Babesia canis vogeli* based on the alignment of *18S rRNA* sequences (using Bab7/Bab9 primer) of the present study together with sequences from different regions and species deposited in GenBank. The tree was constructed using the Kimura-2-parameter model, a maximum-likelihood method. The numbers at the node branches indicate the bootstrap value percentage in 1,000 replicates. *Plasmodium falciparum* is provided as an outgroup species. All *B. canis vogeli* isolates from this study were clustered within a single clade. This clade also comprised *B. canis* *vogeli* sequences from Japan, Brazil, Thailand, India, China, Egypt, the United States, and France, with high bootstrap support. In addition, the tree showed clustering of other *Babesia* subspecies and species, including *B. canis canis*, *B. canis rossi*, and *Babesia gibsoni*, into distinct, separate clades.

The phylogenetic tree generated from Bab7/Bab9-amplified 18S rRNA sequences showed that all *B. canis vogeli* isolates from this study clustered within a single clade. This clade also comprised *B. canis vogeli* sequences from Japan, Brazil, Thailand, India, China, Egypt, the USA, and France, with a high bootstrap support of 99% and 94.15%–100% identity (Table 4). In addition, the tree showed clustering of other *Babesia* subspecies and species, including *B. canis canis*, *B. canis rossi*, and *B. gibsoni*, into distinct, separate clades (Figure 3).

**Table 4:** Pairwise identity (%) among samples from the current investigation and sequences from different locations and species of *B. canis vogeli 18S rRNA* sequences (Bab7/Bab9).

Sample code	1	2	3	4	5	6	7	8	9	10	11	12	13	14	15	16	17
1. *P. falciparum* (M19172)	100																
2. *B. gibsoni*, India (MN134517)	76.79	100															
3. *B. rossi*, Africa (KY463430)	76.08	88.75	100														
4. *B. canis*, China (MK571835)	75.63	91.26	91.00	100													
5. 18 sequences of *B. vogeli* BAB79	77.30	91.73	91.21	95.41	100												
6. *B. vogeli* BAB79-008	73.79	86.34	85.82	90.08	94.64	100											
7. *B. vogeli* BAB79-011	76.59	91.49	90.98	94.66	98.21	92.88	100										
8. *B. vogeli* BAB79-012	77.10	91.49	90.98	95.17	100	94.91	97.96	100									
9. *B. vogeli* BAB79-080	76.59	90.72	90.21	94.40	98.98	94.15	97.20	98.98	100								
10. *B. vogeli*, Thailand (KF621061)	77.90	89.67	88.93	93.84	100	98.91	97.83	100	99.64	100							
11. *B. vogeli*, India (PP859306)	77.35	91.75	91.24	95.42	100	94.66	98.22	99.75	98.98	100	100						
12. *B. vogeli,* Brazil (MN823219)	77.35	91.75	91.24	95.42	100	94.66	98.22	99.75	98.98	100	100	100					
13. *B. vogeli,* China (KJ939326)	77.10	91.49	90.98	95.17	100	94.40	97.96	99.49	98.73	99.64	99.75	99.75	100				
14. *B. vogeli*, Japan (DQ111766)	81.55	87.28	88.16	92.17	100	100	97.82	100	99.56	100	100	100	99.56	100			
15. *B. vogeli*, Egypt (AY371197)	76.84	91.49	90.98	94.91	99.49	94.15	97.71	99.24	98.47	99.28	99.49	99.49	99.24	99.13	100		
16. *B. vogeli*, USA (AY371198)	77.10	91.49	90.98	95.17	100	94.40	97.96	99.49	98.73	99.64	99.75	99.75	99.49	99.56	99.24	100	
17. *B. vogeli,* France (AY072925)	77.10	91.49	90.98	95.17	100	94.40	97.96	99.49	98.73	99.64	99.75	99.75	99.49	99.56	99.24	100	100

*18 samples, including BAB79-001-BAB79-007, BAB79-009, BAB79-010, BAB79-013-BAB79-019, BAB79-054, and BAB79-119. *P. falciparum = Plasmodium falciparum, B. gibsoni = Babesia gibsoni, B. canis = Babesia canis, B. vogeli = Babesia vogeli, B. rossi = Babesia rossi*

**Figure 4:**
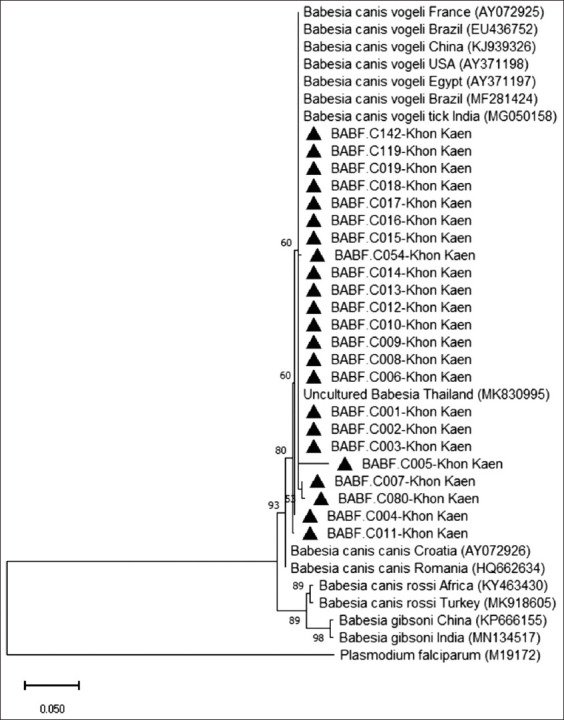
Phylogenetic tree of *Babesia* *canis vogeli* based on the alignment of *18S rRNA* sequences (using Babf/Babc primer) of the present study together with sequences from different regions and species deposited in GenBank. The tree was constructed using the Kimura-2-parameter model, a maximum-likelihood method. The numbers at the node branches indicate the bootstrap value percentage in 1,000 replicates. *Plasmodium falciparum* is provided as an outgroup species. All *B. canis vogeli* isolates from this study clustered with *B. canis vogeli* from Brazil, France, China, the USA, Egypt, India, and Thailand, supported by moderate bootstrap values. As in the previous tree, *B. canis vogeli* formed a distinct cluster that was clearly separated from other *Babesia* subspecies and species, such as *B. canis canis*, *B. canis rossi*, and *Babesia gibsoni*, each of which formed their own clades.

The phylogenetic tree based on Babf/Babc-amplified sequences also showed that all *B. canis vogeli* isolates from this study clustered with *B. canis vogeli* isolates from Brazil, France, China, the USA, Egypt, India, and Thailand, supported by a moderate bootstrap value (60%) and sequence identity ranging from 97.34% to 100% (Table 5). As in the previous tree, *B. canis vogeli* formed a distinct cluster that was clearly separated from other *Babesia* subspecies and species, such as *B. canis canis*, *B. canis rossi*, and *B. gibsoni*, each of which formed their own clades (Figure 4).

**Table 5:** Pairwise identity (%) among samples from the current investigation and sequences from different locations and species of *B. canis vogeli 18S rRNA* sequences (Babf/Babc).

Sample code	1	2	3	4	5	6	7	8	9	10	11	12	13	14	15	16	17	18	19	20
1. *P. falciparum* (M19172)	100																			
2. *B. gibsoni*, India (MN134517)	72.75	100																		
3. *B. canis,* Croatia (AY072926)	73	95.02	100																	
4. *B. rossi,* African (KY463430)	72.43	97.34	96.35	100																
5. Uncultured Babesia, Thailand (MK830995)	73	94.02	99	95.35	100															
6. *B. vogeli* BABFC001	73	94.02	99	95.35	100	100														
7. *B. vogeli* BABFC004	73.24	94.33	99.33	95.7	99.67	99.67	100													
8. *B. vogeli* BABFC005	71.33	91.69	96.35	93.02	97.34	97.34	97	100												
9. *B. vogeli* BABFC007	72.67	93.69	98.67	95.02	99.67	99.67	99.33	97.01	100											
10. *B. vogeli* BABFC011	72.67	94.35	99.34	95.68	99.34	99.34	99.67	96.68	99	100										
11. *B. vogeli* BABFC054	72.67	93.69	98.67	95.02	99.67	99.67	99.33	97.01	99.34	99	100									
12. *B. vogeli* BABFC080	72.33	93.36	98.34	94.68	99.34	99.34	99	97.34	99.67	98.67	99	100								
13. Sixteen sequences of *B. vogeli* BABFC*	73	94.02	99	95.35	100	100	99.67	97.34	99.67	99.34	99.67	99.34	100							
14. *B. vogeli*, India (MG050158)	73	94.02	99	95.35	100	100	99.67	97.34	99.67	99.34	99.67	99.34	100	100						
15. *B. vogeli*, Brazil (MF281424)	73	94.02	99	95.35	100	100	99.67	97.34	99.67	99.34	99.67	99.34	100	100	100					
16. *B. vogeli*, Egypt (AY371197)	73	94.02	99	95.35	100	100	99.67	97.34	99.67	99.34	99.67	99.34	100	100	100	100				
17. *B. vogeli*, USA (AY371198)	73	94.02	99	95.35	100	100	99.67	97.34	99.67	99.34	99.67	99.34	100	100	100	100	100			
18. *B. vogeli*, China (KJ939326)	73	94.02	99	95.35	100	100	99.67	97.34	99.67	99.34	99.67	99.34	100	100	100	100	100	100		
19. *B. vogeli*, France (AY072925)	73	94.02	99	95.35	100	100	99.67	97.34	99.67	99.34	99.67	99.34	100	100	100	100	100	100	100	
20. *B. vogeli*, Brazil (EU436752)	73	94.02	99	95.35	100	100	99.67	97.34	99.67	99.34	99.67	99.34	100	100	100	100	100	100	100	100

* 16 samples, including BABFC002, BABFC003, BABFC006, BABFC008-BABFC010, BABFC012-BABFC019, BABFC142, and BABFC119. *P. falciparum = Plasmodium falciparum, B. gibsoni = Babesia gibsoni, B. canis = Babesia canis, B. vogeli = Babesia vogeli, B. rossi = Babesia rossi*

### Diagnostic performance of the primer sets

The diagnostic performance of the two primer sets was evaluated and compared by calculating sensitivity, specificity, and PPV using PCR and sequencing results. The Bab7/Bab9 primer set demonstrated a sensitivity of 95.65%, whereas the Babf/Babc primer set showed 100% sensitivity for detecting *B. canis vogeli*.

Although *H. canis* was detected in 20/159 (12.58%) dogs using the Bab7/Bab9 primer set, these were not classified as false positives because the amplicon size was distinct from that of *B. canis*. On sequence confirmation, all positive bands observed at 490 bp (Bab7/Bab9) and 394 bp (Babf/Babc) corresponded to *B. canis vogeli*. Therefore, both the Bab7/Bab9 and Babf/Babc primer sets achieved 100% specificity and 100% PPV for diagnosing *B. canis vogeli*. Table 6 presents a summary of these results.

**Table 6:** Evaluation of the sensitivity, specificity, and PPV of each primer set for amplifying the *18S rRNA* gene of *B. canis* from 159 blood samples of dogs.

Primer	Positive		Negative		Diagnostic value					
		
	True	False	True	False	Sensitivity (%)	95% CI	Specificity (%)	95% CI	PPV (%)	95% CI
Bab7/Bab9	22	0	136	1	95.65	78.05–99.89	100	97.30–100	100	84.56–100
Babf/Babc	23	0	136	0	100	85.18–100	100	97.32–100	100	85.18–100

PPV = Positive predictive value, CI = Confidence interval, *B. canis = Babesia canis*

## DISCUSSION

### Epidemiology of canine babesiosis in Thailand

Canine babesiosis is a tick-borne disease that has been documented in several provinces of Thailand, with *B. canis vogeli* being the predominant etiologic subspecies in dogs [8, 33]. The brown dog tick (*R. sanguineus*), which is prevalent throughout the region, is the principal vector of this parasite [22]. Although various diagnostic tools exist for detecting *B. canis vogeli* infection, molecular assays provide greater reliability and sensitivity for identifying and differentiating *Babesia* species [2, 23, 27, 34].

### Molecular detection of the *18S rRNA* gene and primer specificity

The *18S rRNA* gene is commonly used as a DNA marker for eukaryotic detection because its conserved regions support universal primer design, and its variable regions provide valuable phylogenetic information. These features make the *18S rRNA* gene a useful tool for studying Apicomplexan parasites, assessing diversity, and determining phylogenetic relationships [26, 27, 29].

The Bab7/Bab9 primers used in this study are recognized for their ability to amplify a broad range of *Babesia* species [31]. Only *B. canis vogeli* was detected in this investigation, with no evidence of other subspecies such as *B. canis canis* or *B. canis rossi*. The Bab7/Bab9 primer set also amplified *H. canis*, generating a distinct amplicon of approximately 503 bp. Similar PCR cross-reactivity between *Babesia* and *Hepatozoon* species has been reported by O’Dwyer *et al*. [35] and Panda *et al*. [36], who observed unintended amplification when using primers Piro A1 and Piro B for *Babesia* detection.

### Phylogenetic relationships and sequence diversity

Sequence analysis of *B. canis vogeli* *18S rRNA* revealed high similarity to GenBank reference sequences. The phylogenetic tree based on Bab7/Bab9 showed clustering of *B. canis vogeli* isolates with sequences from Brazil, India, China, and Thailand. This clustering reflects high genetic similarity and conserved phylogenetic relationships across geographic regions, whereas *B. canis canis*, *B. canis rossi*, *B. gibsoni*, and *P. falciparum* clustered into their respective subclades. Some sequences, such as BAB7.9/011, showed slight divergence within the *B. canis vogeli* clade, suggesting minor genetic variation.

Babf/Babc-based phylogenetic analysis revealed similar clustering patterns, with sequences from this study grouped with *B. canis vogeli* sequences from Brazil, India, China, and Egypt. Other *Babesia* species and the outgroup *P. falciparum* formed distinct subclades, confirming the clustering specificity. Minor sequence variations were observed among BABF.C005, BABF.C007, BABF.C080, BABF.C004, and BABF.C011, indicating slight intraspecific variation.

Similar low genetic variation has been reported in previous studies of Thai [7] and Malaysian [37] *B. canis vogeli* isolates. Although the *18S rRNA* gene is conserved and evolves slowly, making it useful for species-level identification, it may lack sufficient resolution for detailed intraspecies phylogenetic analysis. Nevertheless, the *18S rRNA* gene remains a valuable tool for species-level identification and classification of apicomplexan parasites [24, 29].

### Clinical relevance of *B. canis* vogeli infection

Canine babesiosis can cause asymptomatic, mild, or severe clinical symptoms, depending on the pathogen’s virulence and the host’s immune status. *B. canis vogeli* is the least virulent pathogen among *Babesia* species, frequently resulting in asymptomatic to mild infections in adult dogs. However, it can also result in moderate to severe clinical disease in young dogs [9, 11, 38].

*B. canis vogeli* infection has been associated with various symptoms, including pyrexia, anorexia, weakness, lethargy, pale mucous membranes, tachycardia, tachypnea, splenomegaly, icterus, and hemolytic anemia [39]. Dogs infected with *B. canis vogeli* in this study exhibited several clinical signs, which were often non-specific and overlapped with symptoms of other vector-borne infections. Because most infected dogs were also coinfected with *E. canis* or *A. platys*, it was difficult to attribute the observed symptoms solely to *B. canis vogeli*.

One dog infected solely with *B. canis vogeli* was asymptomatic and exhibited normal hematological and biochemical values. These findings show that clinical signs alone are insufficient for a definitive diagnosis of babesiosis.

### Diagnostic performance of PCR primer sets

PCR increased the *Babesia* infection detection rate, highlighting its superior sensitivity in detecting low parasitemia, particularly during the early or subclinical stages of infection [21, 40, 41]. The diagnostic accuracy of each primer set was evaluated by calculating sensitivity, specificity, and PPV using sequence-confirmed results as the reference standard.

The Babf/Babc primer set demonstrated perfect diagnostic performance with 100% sensitivity, specificity, and PPV. The Bab7/Bab9 primer set showed slightly lower sensitivity (95.65%) but maintained 100% specificity and PPV, comparable to Babf/Babc. The failure of amplification in one sample using the Bab7/Bab9 primer set may have resulted from primer-template mismatches. As reported by Stadhouders *et al*. [42], such mismatches can reduce the stability of the primer-template duplex and impair polymerase extension, potentially leading to biased amplification or even PCR failure.

Both primer sets shared the same detection limit, successfully identifying *Babesia* DNA plasmids at concentrations as low as 10^5^ copies. However, because the Bab7/Bab9 set also amplified *H. canis*, misinterpretation could occur if amplicon sizes were not carefully differentiated. Thus, although both primer sets demonstrated strong performance, Babf/Babc is preferred for its greater diagnostic accuracy and specificity to *B. canis vogeli*.

### Importance of differentiating *B. canis vogeli* and *H. canis*

Accurate differentiation between *B. canis vogeli* and *H. canis*, especially in cases of coinfection, is clinically important because mixed infections can cause overlapping or unusual clinical signs [1]. *B. canis vogeli* and *H. canis* are important tick-borne diseases in Thailand, sharing the same main vector, *R. sanguineus*, but having different transmission routes. A single tick can carry more than one pathogen at the same time, which increases the chance of coinfection in dogs [10, 12, 34].

*B. canis vogeli* or *H. canis* infection alone can be mild or asymptomatic to severe. When parasite levels are high, both can cause similar signs, including fever, anemia, lethargy, and anorexia [10]. In co-infected dogs, the disease can become more severe and complicated, especially in clinical manifestations, making management more difficult. Therefore, correctly identifying and differentiating these pathogens is important for proper treatment and better outcomes in endemic areas.

### Limitations of the study

This study has several limitations. Due to the small-scale of the dog population, only one dog was found to be infected with *B. canis vogeli*, thus limiting a more comprehensive evaluation of the hematological and biochemical changes. Furthermore, the study relied on the *18S rRNA* gene as the molecular marker, which is useful for species-level identification and phylogenetic analysis, but has limited resolution for detecting intraspecific genetic diversity.

## CONCLUSION

This study provides the first comparative evaluation of two molecular primer sets, Bab7/Bab9 and Babf/Babc, for detecting *B. canis vogeli* in naturally infected dogs from Khon Kaen, Thailand. While microscopic examination identified *B. canis* in 19/159 (11.95%) of samples, PCR increased detection to 23/159 (14.47%), highlighting its superior sensitivity for low-parasitemia and subclinical cases. Both primer sets achieved 100% specificity and PPV, but Babf/Babc demonstrated slightly higher sensitivity (100%) compared to Bab7/Bab9 (95.65%). Sequence analysis confirmed a high degree of genetic similarity between local *B. canis vogeli* isolates and those from Brazil, India, China, and other regions, with phylogenetic clustering supporting conserved global distribution. Notably, Bab7/Bab9 also amplified *Hepatozoon canis*, underscoring the need for careful amplicon size differentiation to avoid misinterpretation in endemic areas with mixed infections.

The results support the use of the Babf/Babc primer set as a preferred diagnostic tool for *B. canis vogeli* in both clinical and epidemiological settings due to its greater diagnostic accuracy and absence of cross-amplification with *H. canis*. Accurate molecular identification enables earlier intervention, targeted treatment, and better control of vector-borne pathogens in endemic regions.

This work combined field-sourced clinical samples, dual-primer molecular screening, sequence confirmation, and phylogenetic analysis, providing both diagnostic performance data and epidemiological insight into *B. canis vogeli* circulation in northeastern Thailand. The inclusion of *H. canis* detection highlights the clinical relevance of differentiating co-circulating pathogens sharing the same tick vector.

The study’s geographic focus on Khon Kaen limits extrapolation to all regions of Thailand. In addition, while *18S rRNA* gene analysis is valuable for species-level identification, its conserved nature restricts resolution for intraspecific diversity, potentially underestimating subtle genetic variations among *B. canis vogeli* strains.

Further studies incorporating larger, multi-regional sample sets and higher-resolution genetic markers such as ITS or mitochondrial genes could improve phylogenetic resolution. Evaluating molecular assays alongside quantitative PCR and point-of-care diagnostics would also enhance surveillance capacity. Longitudinal monitoring could reveal seasonal and vector-related influences on infection dynamics.

Overall, this study advances the diagnostic framework for canine babesiosis in Thailand, demonstrating the value of molecular assays in detecting and characterizing *B. canis vogeli*. Adoption of highly specific primer sets, coupled with vigilant differentiation from *H. canis*, will strengthen both veterinary clinical care and epidemiological surveillance, ultimately contributing to more effective control of tick-borne diseases in endemic regions.

## AUTHORS’ CONTRIBUTIONS

CAPK and NF: Conceived, designed, and coordinated the study. NF: Sampling and data collection. CAPK and NF: Laboratory work and statistical analysis. CAPK: Data analyses and writing-original draft preparation. NF: Writing-review and editing. NF and PP: Supervised the study. All authors have read and approved the final manuscript.
